# Deciphering single-cell genomic architecture: insights into cellular heterogeneity and regulatory dynamics

**DOI:** 10.1186/s44342-025-00037-4

**Published:** 2025-02-11

**Authors:** Byunghee Kang, Hyeonji Lee, Tae-Young Roh

**Affiliations:** 1 Department of Life Sciences, Pohang University of Science and Technology (POSTECH), Pohang, 37673 Republic of Korea; 2 Department of Life Sciences, Ewha Womans University, Seoul, 03760 Republic of Korea

**Keywords:** Genome architecture, Epigenome, Single cell, Sequencing, Mapping

## Abstract

**Background:**

The genomic architecture of eukaryotes exhibits dynamic spatial and temporal changes, enabling cellular processes critical for maintaining viability and functional diversity. Recent advances in sequencing technologies have facilitated the dissection of genomic architecture and functional activity at single-cell resolution, moving beyond the averaged signals typically derived from bulk cell analyses.

**Main body:**

The advent of single-cell genomics and epigenomics has yielded transformative insights into cellular heterogeneity, behavior, and biological complexity with unparalleled genomic resolution and reproducibility. This review summarizes recent progress in the characterization of genomic architecture at the single-cell level, emphasizing the impact of structural variation and chromatin organization on gene regulatory networks and cellular identity.

**Conclusion:**

Future directions in single-cell genomics and high-resolution epigenomic methodologies are explored, focusing on emerging challenges and potential impacts on the understanding of cellular states, regulatory dynamics, and the intricate mechanisms driving cellular function and diversity. Future perspectives on the challenges and potential implications of single-cell genomics, along with high-resolution genomic and epigenomic technologies for understanding cellular states and regulatory dynamics, are also discussed.

## Introduction

In the past decade, the fields of genomics and epigenomics have undergone a profound transformation, largely due to breakthroughs in sequencing technology. Traditional models of genomic function have been redefined as next-generation sequencing (NGS) has progressed to unprecedented resolution, even enabling analysis at the single-cell level. Compared to studies of bulk cell populations, single-cell analysis provides a powerful framework for examining cellular heterogeneity within populations, revealing intricate differences among individual cells in multicellular tissues or organisms. These findings underscore how different cell types within the same tissue or organism perform diverse functions, reflecting a variety of epigenomic landscapes despite a shared genome.

The emergence of NGS has driven traditional genomic studies into deeper and broader parallel dissection of genome at a nucleotide level. These technologies enable the study of DNA and RNA without the need for prior sequence information, allowing for discoveries across unexplored genomic regions. NGS platforms, offering a high-throughput capability, generate extensive data that range from relatively short reads to long reads that can span entire genomes, creating a data-rich environment that supports research across basic and applied sciences, including translational and clinical genomics. Today, genomics permeates every branch of biology and medicine, enabling both foundational research and targeted medical applications.

Single-cell analyses have been particularly revealing, exposing cellular heterogeneity across major omics layers, including transcriptomics, epigenomics, and genomic variation. For instance, single-cell RNA sequencing (scRNA-Seq) allows detailed examination of transcriptomic profiles in individual cells, which helps to map tissue structure and dynamics by identifying both known and novel cell types. Distinctions between cell types—despite identical genomic content—are often rooted in epigenomic variations, which include DNA and histone modifications, chromatin accessibility, and 3D genome architecture. Single-cell epigenomic assays thus play a critical role in identifying functional regulatory elements and chromatin factors, providing insights into cell-specific regulatory landscapes [1]. Cell type-specific genomic variations such as single-nucleotide variations (SNVs) and copy-number variations (CNVs) can be identified using single-cell whole-genome amplification, offering another layer of understanding into cellular diversity [2].

International initiatives, such as ENCODE, the Roadmap Epigenome Project, the International Human Epigenome Consortium, EpiGeneSys, FANTOM, and the 4D Nucleome (4DN) project, have contributed to a genome-wide annotation of genes and regulatory elements, offering profound insights into nuclear architecture, dynamics, and function. Specifically, the 4DN project, launched by the National Institutes of Health (NIH) in 2014, aims to map chromosome organization in time and space and to elucidate how chromatin structure affects gene regulation in both cell populations and individual cells in humans and mice. The 4DN project integrates 3D genomics technologies, single-cell sequencing, high-resolution microscopy, and bioinformatics to track temporal changes in 3D genome structure, ultimately advancing our understanding of chromatin dynamics and their regulatory roles [3].

To capture the dynamics of individual cells, spatial gene expression data obtained from scRNA-Seq can be combined with visualization techniques like RNA in situ hybridization (ISH), live-cell fluorescence microscopy, or antibody-based methods, allowing the spatial localization and quantification of gene expression within the nucleus. These integrated approaches validate cellular responses, physical phenotypes, and subnuclear localization, providing a comprehensive view of cell state and behavior [4].

In this review, we present recently developed mapping techniques designed to resolve genome structure at high resolution and to elucidate genome function at the single-cell level. We highlight the latest methodologies and their applications in single-cell genomics and provide an overview of the advancements and challenges in understanding genomic and epigenomic architecture as it relates to cellular heterogeneity.

## Mapping the genome structure in space and time

The nucleus contains distinct, membrane-free nuclear structures and compartments, including various nuclear bodies. The genome-wide three-dimensional organization of chromosomes can be elucidated using proximity ligation techniques followed by high-throughput sequencing [5]. The high-resolution chromosome conformation capture technique (Hi-C), an advanced version of the 3C method, quantifies all-to-all interactions between genomic loci, enabling detailed mapping of spatial genome organization, such as chromatin folding [5, 6]. The Hi-C genome map confirmed the spatial compartmentation such as chromosome territories, gene-rich euchromatin, and gene-poor heterochromatin. The nucleus can be subdivided into smaller structural components conceptually such as open/closed chromatin or A/B compartments [5], topologically associated domain (TADs) [7, 8], and chromatin loops [9].

Considering long-range physical interactions, transcriptional states of a genome were defined by active A compartment and inactive B compartment. The A/B compartments were estimated by an eigenvector analysis of the genome contact matrix after normalization. Megabase-scale regions of local chromatin interactions, termed “topological domains” or TADs, are bounded by sites enriched with CTCF and cohesin proteins, which facilitate chromatin structure and regulatory interactions [9]. Enhancer-mediated transcriptional control can be explained by chromatin contact with target genes by forming consistent loops within a TAD. Compartments and TADs are highly conserved across species and identified robustly among Hi-C replicates. Chromatin loops, which play a key role in genome regulation, vary in detection sensitivity depending on sequencing depth, resolution, and noise levels in Hi-C datasets. High sequencing depth is especially critical for accurate loop detection, ensuring that finer-scale interactions within the genome are robustly captured and analyzed [10].

**Fig. 1:**
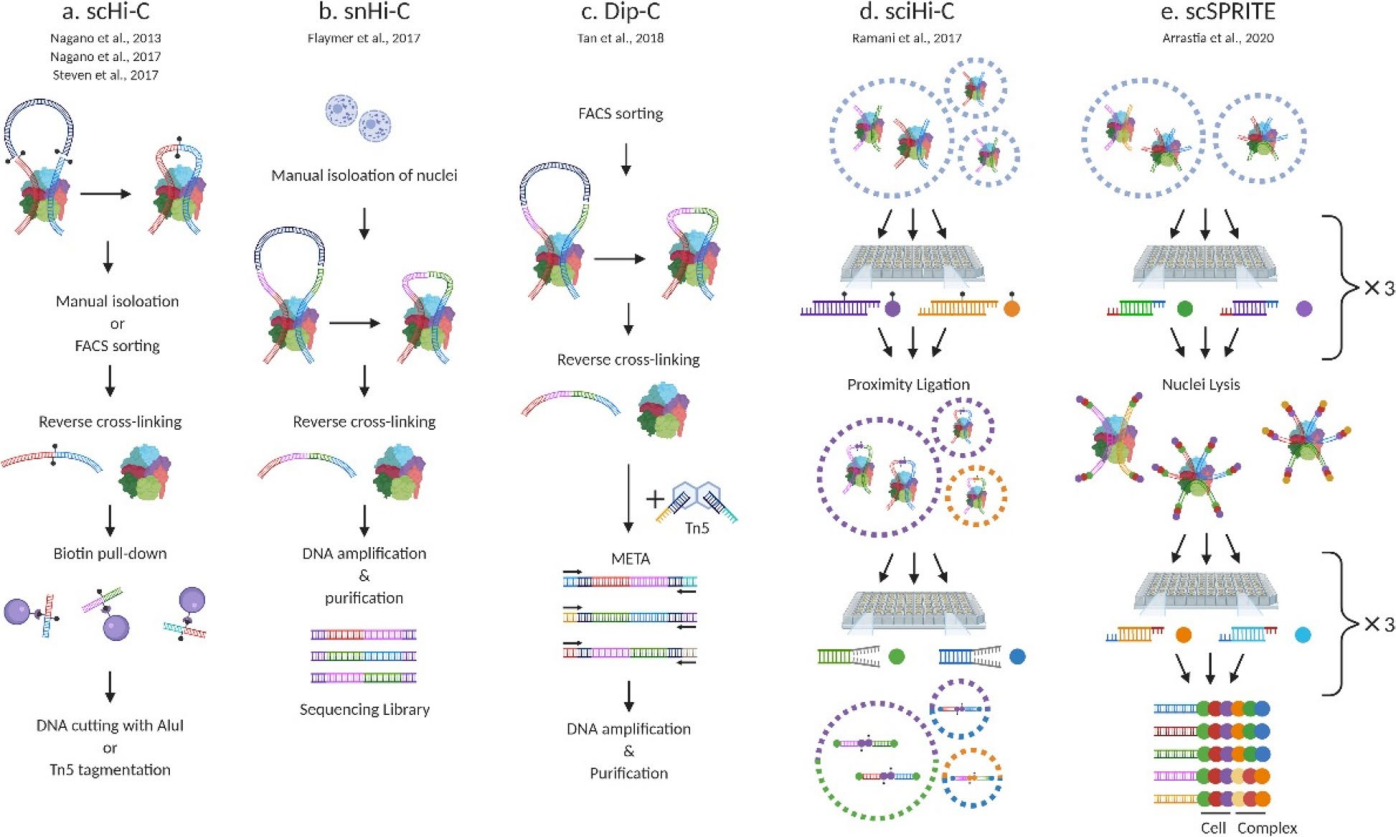
Various single-cell analysis methodologies for chromosome conformation. **a** scHi-C. **b** snHi-C. **c** Dip-C. **d** sciHi-C. **e** scSPRITE

In single-cell Hi-C, interactions between genomic loci exhibit high variability across individual cells, resulting in sparse and noisy datasets. However, pooled single-cell Hi-C data shows similar TADs with that of bulk Hi-C [11]. This suggests that the chromatin structure of the individual cell is dynamic but also has commonality considering the loss of contact information due to limitation of proximity ligation. Additionally, single-cell Hi-C (scHi-C) (Fig. 1a) showed that the information of localization of active gene domains to boundaries of chromosome territory bridges the genomics and microscopic imaging [11, 12]. To extract the meaningful information from the sparse and noisy scHi-C data, several computational and experimental approaches have been developed. For instance, a natural language processing method called topic modeling was applied to differentiate cell-cycle stage-specific and tissue-specific cells [13]. Another recent report revealed single-nucleotide polymorphism variability and distinct structure of imprinted loci between two alleles by Dip-C method using single diploid human cells at 20-kb resolution [14]. An integrative analysis of DNA methylation and chromatin architecture in single cells (scMethyl-HiC) has been proposed to elucidate simultaneous DNA methylome and chromatin loops [15].

However, three-dimensional organization in the nucleus does not satisfy the need for comprehensive understanding of four-dimensional information including nuclear dynamics across time. In single-cell Hi-C, capturing the temporal dynamics of chromatin interactions poses a significant challenge. Sampling cells at distinct time points enable the reconstruction of temporal changes in chromatin organization, such as the progression of TADs, enhancer-promoter interactions, and chromatin loop dynamics. An alternative approach is pseudo-time analysis, which computationally orders single-cell chromatin interaction profiles along a trajectory representing biological processes, such as differentiation or cell-cycle progression [16]. Studies in mouse embryonic stem cells have demonstrated that chromosomes condense in preparation for mitosis and rapidly expand during early G1 phase, while the genome remains dynamically folded without stable organization throughout interphase. Advanced computational models and machine learning algorithms can enhance these analyses by reconstructing temporal trajectories and uncovering sequential events from static single-cell snapshot datasets [17, 18].

As mentioned in the previous section, the stochastic fluctuation of genomic functions can be measured at multiple time points in individual cells. Integrating multidisciplinary technologies enabled us to easily understand the function of diverse genome structure in the nucleus. The hierarchical 3D genome structure has been interpreted as the following approaches in the 4DN project: identification of chromatin contacts and topologically associating domains (TADs) based on chromatin conformation capture (3C) methods and genome-wide mapping of open and closed chromatin domains [19]. The 4DN project integrates experimental mapping techniques, computational modeling approaches, and functional validation through CRISPR/Cas9-mediated perturbation experiments. This comprehensive network has provided valuable insights into the spatiotemporal organization of the genome and its functional dynamics across various cell types and conditions, including analyses at the single-cell level.

## Sequencing technologies for single-cell analysis

Multiple technologies for measuring genomic architecture at the single-cell level have been modified or optimized from methods originally developed for bulk-cell analysis. For example, the 3C-based methods are the most frequently used as one of genomic approaches to map one-to-one, one-to-all, and/or all-to-all chromatin interactions using large number of cells. In the Hi-C protocol, cells are cross-linked with agents like formaldehyde, and chromatin-bound DNA fragments are isolated for sequencing. This process involves several steps: DNA-end modification, labeling, ligation, and amplification to construct sequencing libraries. The spatially interacting DNA fragments are then mapped onto a reference genome, enabling the construction of a proximity map with megabase resolution. The spatial separation of open and closed chromatin is identified as two genome-wide compartments of dynamic genomic conformations by calculating the genome-wide ensemble average of the interactions. The Hi-C should also provide a spatial information of regulatory elements such as enhancers, silencers, and insulators. Although Hi-C is a powerful tool for elucidating genome organization including interchromosomal contacts, it primarily provides the information of cells as a whole rather than heterogeneous chromatin structure of individual cells.

Accordingly, the single-cell analysis was developed to dissect the underlying complexity in bulk cells, but there are still several limitations. The leverage of single-cell analysis is to provide insights into characterization of heterogeneous cell population and identification of rare cell types. To overcome the limited information from single cells, multiple approaches have been evolved. One example of decoding chromatin organization in a single cell is as follows: a single cell or nucleus is isolated manually or by using flow cytometry and cross-linked. The purified DNA fragments are biotin-labeled and ligated with adapters including specific barcodes for originating cell identification. The topologically associating domains (TADs) and loops are analyzed from the sequence reads [16, 20]. The modified single-nucleus Hi-C (snHi-C) analysis (Fig. 1b) enabled production of 10 times more chromatin contacts per cell comparing with the previous single-cell Hi-C (scHi-C) result [11, 20]. This split-and-pool library preparation method was designed to efficiently label and track individual nuclei [21, 22]. The nuclei are randomly split into 96 wells each containing a unique biotinylated barcode sequence. Barcoded DNA from these wells is pooled, diluted appropriately, and then split into another set of 96 wells. This process is repeated multiple times, generating unique combinations of barcodes with each iteration. The combinatorial synthesis approach dramatically increases the number of possible barcode combinations, allowing for high-throughput sample processing with minimal overlap or ambiguity. As a result, the sequential split-and-pool method effectively ensures the diversity of library and accurate identification of individual nuclei. However, there are issues on some false-positive reads from self-ligated or un-ligated fragments due to inefficient restriction enzyme digestion and ligation [6]. Another issue in a single-cell analysis is that multiple contact map can be hardly obtained because one end of DNA fragment should have only a single chance of ligation with another one for the proximity ligation. Thus, each single-cell Hi-C map leads only to be a part of complete simultaneous interactions in cells.

An improved chromatin conformation capture method, termed diploid chromatin conformation capture (Dip-C) (Fig. 1c), could be helpful to reduce false positives derived from inefficient digestion and ligation [14]. Dip-C technique utilizes high-coverage whole-genome amplification with multiplex end-tagging amplification (META), replacing with processes like biotin-pulldown and blunt-end ligation. By this way, the number of contacts per single cell in Dip-C reaches about 1 million. Ligation-free approaches such as “split-pool recognition of interactions by tag extension” (SPRITE) and chromatin-interaction analysis via droplet-based and barcode-linked sequencing (ChIA-Drop) could be alternative solutions for overcoming insufficient contact information derived from single cells [23, 24]. These methods label crosslinked chromatin with barcodes to detect complete contact maps without proximity ligation. SPRITE uses a split-and-pool synthesis strategy to barcode with odd and even tags that prevent self-ligation and specify chromatin structure. ChIA-Drop adopts droplet microfluidics to separate each chromatin structure into a droplet and capture multiplex chromatin interactions at a single-molecule level. Since one droplet contains one chromatin complex and one barcode, crosslinked chromatin is selectively tagged by the barcode. Similar to single-cell combinatorial indexed Hi-C (sciHi-C) (Fig. 1d), a method that applies combinatorial cellular indexing to chromosome conformation capture [21], single-cell SPRITE (scSPRITE) (Fig. 1e) has been also developed for the measurements of genome-wide maps of 3D DNA structure in thousands of individual nuclei at a time [22].

Advancements in technology are essential to deepen our understanding of higher-order chromatin interactions and their roles in gene regulatory mechanisms at single-cell resolution. Developing and optimizing bioinformatic pipelines are critical for integrative analyses of single-cell Hi-C data in conjunction with other single-cell modalities, such as RNA-seq, DNA methylation profiling, histone modifications, protein binding, chromatin accessibility, and DNA mutation data. Addressing challenges related to low coverage and limited contact information will improve the precision of cell type identification and regulatory analysis. Simultaneous profiling of chromatin structure alongside other genomic or epigenomic features within individual cells holds significant potential, allowing the observation of phased characteristics from a single assay to capture a more comprehensive view of cellular function and regulation.

Recently, several single-cell multi-omics techniques were emerged to measure multiple features in the same cells or nuclei [25–29]. Multi-omics single-cell analysis can provide critical insights into individual cell behavior as shaped by epigenetic modifications and gene expression changes during development and in disease contexts.

## Studies on single-cell-based genome architecture

### Unique chromatin organization of single cell

3D genome organization is expected to be different in individual cells. The key biological question to be addressed is how epigenetically distinct genomes are spatially and temporally established during development and differentiation. Embryos present unique challenges for biological research due to their limited number of cells and the extraordinary complexity of their cell types. Some cells exhibit pluripotency and are distributed in spatially and temporally dynamic patterns during development. Traditional bulk analysis methods struggle to capture the epigenetic and transcriptional landscapes of embryonic cells, as they require larger populations that embryos cannot provide. Single-cell analysis emerges as an indispensable approach in this context, allowing researchers to dissect the intricate chromatin interactions and epigenetic modifications occurring at the level of individual cells. By focusing on single-cell methodologies, it is possible to unravel how distinct genomic and epigenetic profiles are established and regulated during embryonic development, providing critical insights into fundamental biological processes that are otherwise masked by population averaging [30].

Combining imaging with an improved Hi-C protocol, the whole-genome structures of single G1-phase haploid mouse embryonic stem (ES) cells were determined at the 100-kb scale [31]. Folding of chromosomes into TADs and CTCF/cohesin loops does form in only a proportion of cells. The gene network driven by pluripotency factor and nucleosome remodeling deacetylase (NuRD) can be understood from the single-cell genome-wide analysis of 3D interactions of individual regulatory elements and genes. Tachibana-Konwalski group developed single-nucleus Hi-C (snHi-C) and efficiently observed the spatial reorganization of chromatin during transition from transcriptionally active immature oocyte to zygote transition [20]. During oocyte maturation, the average signal strength of TAD, loop, and compartment were significantly decreased, probably due to transcriptional silencing and chromatin restructuring. Because the maternal and paternal genomes are formed by different biological process and possess unique epigenetic features, it should be determined whether their inherited chromatin architecture would be maintained or not after fertilization. Distinct chromatin architecture in haploid nuclei of zygotes was identified; A/B compartmentalization was detected in paternal nuclei but not in maternal nuclei. Also, mature oocyte showed longer range contacts and less cell-to-cell variability compared to immature oocyte. These result showed that zygotic nuclei is different from interphase cells in terms of spatiotemporal chromatin organization [20].

A similar study showed that cohesin-dependent loop extrusion generates higher-order chromatin structures within the one-cell embryo [32]. Cohesin is crucial for the establishment of chromatin loops, TADs, and other large-scale zygote-specific structures, but not compartments in one-cell embryos. The cohesion ring can be released by Wapl, and its deletion leads to increase of the formation of loop and TAD but decrease of the compartmentalization. Furthermore, cohesin limits interchromosomal interactions by compacting chromatin and inactivation of cohesin diminishes differences in loop strengths between the maternal and paternal genomes.

Super-resolution chromatin tracing, using techniques like 3D stochastic optical reconstruction microscopy (STORM) and fluorescence in situ hybridization (FISH) [33], shows chromatin conformation at kilobase- and nanometer-scale resolution. These imaging techniques demonstrate spatially segregated TAD-like domains with distinct boundaries in single cells, emphasizing the persistence of TAD-like structures at the single-cell level. During cell differentiation, TAD partitioning becomes more variable, with heterogeneous structures favoring intra-TAD over inter-TAD interactions.

### Cell cycle and replication

Chromosomes during cell cycle exhibit dynamic structural changes from highly condensed mitotic structures to decondensed interphase structures. Population-based Hi-C data shows an averaged chromatin contact information from cells in different cell cycle phases, but cell-cycle phasing information of single nuclei can provide an insight on the cell-cycle dynamics of chromosomal structural features. Multiplexed high-resolution single-cell Hi-C using flow cytometry sorting enables continuous chromatin structure profiling throughout the cell cycle. To phase single cells at various stages of the cell cycle, comparative analysis of short-range (< 2 Mb) versus mitotic band (2–12 Mb) contacts per cell showed a gradual chromosome reorganization as mouse embryonic stem cells undergo mitosis [16]. The localized loops are enriched after mitosis and maintained through G1 phase, but aggregated contacts at early- and late-replicating regions are decreased in early-S phase and late-S phase for each. There is higher loop enrichment at pre-M phase than post-M phase. DNA replication is associated with establishment of compartments and a reduction in TAD insulation. TAD boundaries disappear after the G1 phase, and the chromatin contacts are increased across TAD boundaries. On the contrary, compartments show an increase as the cell cycle progresses and the highest enrichment at the end of the S phase. Additionally, distributions of chromosomal conformations are preferentially localized in nucleolar organizer regions by 3D whole-genome modeling and analysis. This indicates that TAD insulation is associated with the replication and exists as stable independent entity [34].

TAD boundaries positively correlate with replication domain boundaries, whereas within a cell type adjacent TADs replicate at similar times with a little variation [35]. Lamina is associated with late-replicating regions and replication-timing transitions between early and late replication. Most replication timing transition regions (TTRs) are overlapped with late-replicating regions with no discontinuity at late TTR borders, whereas early TTR borders indicate the structural boundaries of replication domains. Two TAD classes are grouped by unsupervised clustering of TADs such that class A corresponded to early TADs, whereas class B corresponded to TADs within either TTRs or late regions. It is expected that TADs may switch replication timing by obtaining features associated with their new subnuclear compartment.

Single-cell replication profiling measured by DNA copy number demonstrates that borders between replicated and unreplicated DNA are highly conserved across cells, indicating active and inactive compartments of the nucleus [36]. Intrinsic variability and extrinsic cell-to-cell variability are similar between cells, between homologs within cells, and between all domains, regardless of the replication timing or chromatin state. Replication timing has less stochastic variation than selection of replication origin.

### 3D modeling and computational approaches for chromatin structure

Quantitative models of nuclear organization in diverse cell types and conditions have great interest for mechanistic interpretation of experimental observations. Two major computational approaches for modeling genome architecture can be made; data-driven computation uses experimental data like Hi-C-based sequencing data and imaging data to construct an assembly of chromatin interaction maps from contact information. Another approach is de novo modeling which first creates a predictive ensemble of chromatin conformations from hypothesis and then tests with experimental evidences [3].

With single-cell chromatin structure data, it is possible to investigate heterogeneity of 3D models generated from individual cells. Intrachromosomal contacts can be used as distance restrains, and random model can be fitted with given genomic window to best explain contact matrix. However, generating accurate model from 3C-based assays is a challenge since it is hard to distinguish chromosome homology information from diploid data. To overcome this issue, some modeling studies can be performed by using X chromosome [11] or haploid cells [16, 31].

The variation in chromosome structure between different cell types is identified by computational approaches using sparse and heterogeneous single-cell Hi-C data. A population-based probabilistic approach for deconvoluting Hi-C data into a model population of distinct diploid 3D genome structures was proposed to identify chromatin interactions likely to coexist in individual cells [37]. Using this algorithm by maximum likelihood estimation, chromosome-specific clusters are found to play a key role in the overall chromosome positioning in the nucleus and stabilizing specific chromatin interactions.

To extract meaningful insights from single-cell Hi-C data, a variety of pipelines and tools have been developed (Tables 1 and 2). Single-cell Hi-C preprocessing and normalization tools tackle key challenges such as data sparsity, biases, and noise, but they differ considerably in their methodologies. For preprocessing, nuc_processing [31] and hickit [14] prioritize generating high-resolution datasets, with nuc_processing targeting nucleosome-level granularity and hickit optimized for efficient data handling in large-scale experiments. Tools like GiniQC [38] specialize in quality control, using Gini coefficients to identify data variability and ensure reliability. In normalization, BandNorm [39] corrects systematic biases in interaction matrices, such as sequencing depth and chromatin accessibility, while scHiCNorm [40] emphasizes normalization tailored for highly sparse single-cell datasets, enhancing interpretability for downstream analyses. Meanwhile, SnapHiC [41], and its enhanced version SnapHiC-D [42], uniquely integrate spatial chromatin data to refine interaction maps, with specific focus on enhancer-promoter loops. Together, these diverse tools provide a robust foundation for accurate and high-quality data processing.

**Table 1:** Pipelines for single-cell chromatin structure sequencing data

Name	Purpose	Targeting approach	Ref	Language	Web page
NucProcess	Pipeline for scHi-C analysis	scHi-C	[31]	Python	https://github.com/TheLaueLab/nuc_processing
combinatorialHiC	Pipeline for sciHi-C analysis	sciHi-C	[21]	Python	https://github.com/VRam142/combinatorialHiC
scHiC	Pipeline for scHi-C analysis	scHi-C	[16]	R and Perl	https://github.com/tanaylab/schic2
Dip-C	Pipeline for Dip-C analysis	Dip-C	[14]	Python	https://github.com/tanlongzhi/dip-c
Hickit	Pipeline for Dip-C analysis	Dip-C	[14]	C	https://github.com/lh3/hickit
scHiCTools	Computational toolbox for analyzing scHi-C data	scHi-C	[43]	Python	https://github.com/liu-bioinfo-lab/scHiCTools
SnapHiC	Pipeline for scHi-C analysis	scHi-C	[41]	Python	https://github.com/HuMingLab/SnapHiC
SnapHiC2	Pipeline for scHi-C analysis	scHi-C	[44]	Python	https://github.com/HuMingLab/SnapHiC/releases/tag/v0.2.2
scDEC-Hi-C	Comprehensive single-cell Hi-C data analysis	scHi-C	[45]	Python	https://github.com/kimmo1019/scDEC-Hi-C

**Table 2:** Computational tools developed for scHi-C analysis

Name	Purpose	Technique	Targeting approach	Ref	Language	Web page
MBO	Inference 3D structure of chromosomes from single-cell Hi-C	Manifold-based optimization (MBO)	scHi-C	[46]	MATLAB	https://folk.universitetetioslo.no/jonaspau/mbo/
NucDynamics	Inference 3D structure of chromosomes from single-cell Hi-C	Force field approach integrating molecular dynamics and optimization	scHi-C	[31]	Python and Cython	https://github.com/TheLaueLab/nuc_dynamics
Lavaburst	Contact cluster identification	Network modularity score	scHi-C	[20]	Python	https://github.com/nvictus/lavaburst
scHiCNorm	Normalization of scHi-C data	Poisson and negative binomial model	scHi-C	[40]	R and Perl	http://dna.cs.miami.edu/scHiCNorm/
HiCRep + MDS	Embedding scHi-C data into two dimensions	HiCRep and multidimensional scaling (MDS)	scHi-C	[40]		
scHiCluster	Clustering cell type-specific chromosome structural patterns	Linear convolution and random walk	scHi-C	[47]	Python	https://github.com/zhoujt1994/scHiCluster
SCL	Inference 3D structure of chromosomes from single-cell Hi-C	Cubic lattice representation of a chromosome 3D structure and contact matrix imputation based on a 2D Gaussian function	scHi-C	[48]	C++	http://dna.cs.miami.edu/SCL/
SIMBA3D	Inference 3D structure of chromosomes from single-cell Hi-C	Bayesian approach with bulk Hi-C prior and multiscale optimization	scHi-C	[49]	Python	https://github.com/nerettilab/SIMBA3D
GiniQC	Quantifying unevenness in the distribution of interchromosomal reads in the scHi-C contact matrix	Gini coefficient from cumulative distribution of *trans* read pairs	scHi-C	[38]	Python	https://github.com/4dn-dcic/GiniQC
Inter-chromosomal-interactions	Analyzing interchromosomal interaction of single-cell Hi-C data	Significant interchromosomal interactions were derived with assumption where *trans* in single cell follows Bernoulli trial	scHiC	[50]	Python	https://github.com/bignetworks2019/Inter-chromosomal-interactions
schic-topic-model	Distinguishing cell type differences	Latent Dirichlet allocation (LDA)	scHi-C	[13]	Python and R	https://github.com/khj3017/schic-topic-model
deTOKI	TAD-like domain (TLD) detection in single cell	Nonnegative matrix factorization (NMF)	scHi-C	[51]	Python	https://github.com/lixiaoms/TOKI
Si–C	Inference 3D structure of chromosomes from single-cell Hi-C	Bayesian theory framework and polymer physics	scHi-C	[52]	C	https://github.com/TheMengLab/Si-C/tree/master
DPDchrom	Inference 3D structure of chromosomes from single-cell Hi-C	Dissipative particle dynamics (DPD)	scHi-C	[53]	Fortran and Python	https://github.com/polly-code/DPDchrom
Higashi	Multiscale and integrative scHi-C analysis	Transformation of scHi-C data into a hypergraph and hypergraph neural network	scHi-C	[54]	Python	https://github.com/ma-compbio/Higashi
Fast-Higashi	Ultrafast and interpretable scHi-C analysis	Tensor decomposition and partial random walk	scHi-C	[55]	Python	https://github.com/ma-compbio/Fast-Higashi
BandNorm	Normalization of scHi-C data	Baseline scaling-based normalization	scHi-C	[39]	R	https://github.com/keleslab/BandNorm
scVI-3D	Deep generative model for scHi-C data	Nonlinear latent factor model and hierarchical generative model	scHi-C	[39]	Python	https://github.com/keleslab/scVI-3D
HiCS	Association between regulatory factor binding and chromatin domain	Hierarchical chromatin domain structure identification	scHi-C	[56]	Python	https://github.com/YusenYe/HiCS
scHi-Csim	Simulation of scHi-C data	Interval sampling of raw scHi-C data	scHi-C	[57]	Python	https://github.com/zhanglabtools/scHi-CSim
ScHiCEDRN	Imputation of single-cell Hi-C	Generative adversarial network (GAN)	scHi-C	[58]	Python	https://github.com/BioinfoMachineLearning/ScHiCEDRN
DeDoc2	TAD-like domain (TLD) detection in single cell	Seeking minimal structural entropy	scHi-C	[59]	Java	https://github.com/zengguangjie/deDoc2
SnapHiC-D	Differential chromatin contact from scHi-C	Random walk with restart (RWR) and two-sided two-sample *t*-test with Benjamini–Hochberg procedure (FDR)	scHi-C	[42]	Python	https://github.com/HuMingLab/SnapHiC-D
HiC-SGL	Imputation of single-cell Hi-C	Subgraph extraction and graph representation learning	scHi-C	[60]	Python	https://github.com/zhengjh39/HiC-SGL

For improving data quality, tools like Higashi and Fast-Higashi [55] exemplify advanced reconstruction methodologies. Higashi [54] employs deep learning to impute missing interactions and denoise sparse datasets, enabling the extraction of biologically meaningful features such as TADs. Fast-Higashi extends this capability with increased computational efficiency, making it suitable for large-scale datasets. In contrast, tools like deDoc2 [59] aim to identify TAD-like structures (TLSs) by optimizing 2D structural entropy, focusing on the detection of chromatin boundaries. SnapHiC takes a different approach by enhancing chromatin interaction maps through spatial integration, prioritizing localized features like loops and domains. Together, these tools address different aspects of data enhancement, from global imputation to domain-specific refinement, showcasing a range of approaches to improve single-cell Hi-C data quality.

For dimensionality reduction, clustering, and visualization, tools like schic-topic-model [13] and deTOKI [51] provide interpretable embeddings of high-dimensional chromatin interaction data, using natural language processing and geometric methods, respectively. In clustering, scHiCluster [47] groups cells based on chromatin interaction profiles, while scDEC-Hi-C [45] applies deep learning to uncover latent structures in the data. For visualization, MBO [46] and Inter-chromosomal-interactions [50] are tools for exploring chromatin organization. MBO specializes in examining intrachromosomal domains, while Inter-chromosomal-interactions focuses on cross-chromosome contacts. Tools like scVI-3D [39] integrate dimensionality reduction with 3D modeling, bridging the gap between clustering and spatial chromatin analysis. These tools highlight the diverse analytical methods designed to address varying scales and resolutions of chromatin interaction data.

Simulation and modeling tools, such as scHi-CSim [57] and ScHiCEDRN [58], address complementary needs for single-cell Hi-C studies. scHi-CSim simulates synthetic datasets for benchmarking computational pipelines, providing controlled scenarios to evaluate tool performance. In contrast, ScHiCEDRN integrates machine learning to model chromatin dynamics and predict structural variations. For 3D genome reconstruction, tools like SIMBA3D [49], Si–C [52], and DPDchrom [53] focus on reconstructing chromatin architecture and modeling spatial dynamics. HiC-SGL [60] stands out with its advanced graph-based learning approach, predicting sparse chromatin interactions with high precision. Additionally, NucDynamics [31] integrates chromatin interaction data with epigenomic features, offering a multi-omics perspective. These tools represent the forefront of 3D genome research, bridging simulation, prediction, and experimental validation to enhance our understanding of chromatin organization and dynamics.

Studies on single-cell-based genome architecture have been instrumental in advancing our understanding of the spatial organization of chromatin within individual cells, revealing insights into cellular heterogeneity and regulatory complexity. To facilitate this, pipelines for processing and analyzing single-cell chromatin structure sequencing data have been developed, enabling high-resolution assessments of chromatin interactions and structural variations on a per-cell basis (Table 1). Furthermore, computational tools specifically designed for single-cell Hi-C (scHi-C) analysis have emerged, offering robust frameworks for mapping chromatin contact dynamics, uncovering topological domains, and elucidating cell-specific regulatory landscapes (Table 2). Together, these advancements are driving forward the field of single-cell genomics and enhancing our understanding of genome architecture at an unprecedented level of detail.

## Perspective

A key objective in single-cell genomics is to elucidate how genome and epigenome structures regulate cellular function in both health and disease contexts. Achieving this goal has driven extensive research into developing techniques and protocols, including single-cell isolation, library preparation, high-throughput sequencing methods, data processing, mapping algorithms, computational model development, and data interpretation and visualization. For example, the 4D Nucleome (4DN) Project exemplifies how genomic architecture can be understood in terms of chromatin contacts, loops, compartments, cooperativity, heterogeneity, and dynamics by integrating 3D genomics technologies, single-cell sequencing, high-resolution microscopy, and bioinformatics.

There are multiple challenges and promises of current approaches [2, 3, 29, 61, 62]. Despite significant advances, current approaches face several key challenges and hold both promise and limitations. First, while a wide range of methodologies exist for analyzing genome architecture, contact distances, and frequencies, they often lack the resolution and scalability required for high-resolution, large-scale views of dynamic chromatin structures, presenting considerable complexity in their use. Computational modeling has therefore become essential for interpreting and predicting chromatin dynamics based on fragmented structural data. Second, most existing genome architecture models are static, underscoring the importance of temporal dynamics from diverse biological systems to incorporate a unified and dynamic framework. Third, single-cell data generation remains in a developmental stage due to technical limitations in capturing comprehensive genomic information within individual cells; relying on data from portions of a single cell can obscure intrinsic cellular heterogeneity. Fourth, current models should be expanded to include functional mechanisms, such as epigenetic modifications, enhancer-promoter interactions, and transcription factor binding, to provide a comprehensive representation of genome regulation. Lastly, it is essential to rigorously validate experimental and computational models to ensure that inferred mechanisms are applicable across systems and capable of predicting the sequence-structure–function relationship in broader biological contexts.

## Conclusions

Over the past decade, advancements in sequencing technologies and computational resources for single-cell analysis have significantly matured, resulting in an extensive accumulation of data. Single-cell genomics and epigenomics have unveiled cell-to-cell heterogeneity in gene expression, chromatin conformation, accessibility, histone modifications, and DNA methylation at unprecedented resolution. Notably, the 3D organization of genomic architecture has been shown to play a critical role in various biological processes, including fertilization, development, differentiation, and cell division. Structural variations in topologically associating domains (TADs), chromatin loops, and compartments in diverse cell types and conditions have been linked to transcriptional regulation, chromatin remodeling, and differential regulation of cellular functions.

Although insights into the regulation of 3D chromatin conformation are emerging, single-cell genomics and epigenomics still face challenges in achieving comprehensive mechanistic understanding due to current technical limitations. Multi-omics approaches at the single-cell level have the potential to integrate multiple regulatory factors within individual cells, thus providing a more cohesive view of the determinants of cellular identity. Furthermore, rapid advancements in technology are enabling increasingly detailed profiles of individual cellular states, enhancing our understanding of spatiotemporal dynamics in single cells and contributing to a deeper grasp of nuclear processes at the single-cell level.

## Data Availability

No datasets were generated or analysed during the current study.
